# Supplementary material for: Management of Osteoarthritis and Joint Support Using Feed Supplements: A Scoping Review of Undenatured Type II Collagen and Boswellia serrata
Source: Animals (Basel). 2023 Feb 27;13(5):870. doi: 10.3390/ani13050870 (PMC10000124; doi:10.3390/ani13050870)
Supplement: Supplementary file 1 [file animals-13-00870-s001.zip › animals-2145053-supplementary.pdf]

**Table S1.** Description of the feed supplements used in each of the studies

| | Ref. | Complementary feed |
|---|---|---|
| 1 | [55] | UC-II® 1 mg/day (Group II) or 10 mg/day (Group III) of active ingredient (InterHealth Nutraceuticals Inc.) |
| 2 | [30] | UC-II® 10 mg/day (Group II) of active ingredient, Glucosamine 2000 mg/day, Chondroitin 1600 mg/day (Group III), UC-II® 10 mg/day of active ingredient, Glucosamine 2000 mg/day, Chondroitin 1600 mg/day (Group IV) (InterHealth Nutraceuticals Inc.) |
| 3 | [56] | UC-II® 10 mg/day of active ingredient (Group II), (-)-hydroxycitric acid 1800 mg/day (Group III), (-)-hydroxycitric acid 1800 mg/day, chromemate 100 mcg/day, (Group IV), UC-II® 10 mg/day of active ingredient, (-)-hydroxycitric acid 1800 mg/day, chromemate 100 mcg/day (Group V) (InterHealth Nutraceuticals Inc.) |
| 4 | [57] | UC-II® 10 mg/day of active ingredient (InterHealth Nutraceuticals Inc) (Group II) |
| 5 | [58] | UC-II® 10 mg/day of active ingredient (Group II), Glucosamine 2000 mg/day, Chondroitin 1600 mg/day (Group III), UC-II® 10 mg/day of active ingredient, Glucosamine 2000 mg/day, Chondroitin 1600 mg/day (Group IV) (InterHealth Nutraceuticals Inc.) |
| 6 | [59] | Undenatured Type II Collagen 10 mg/day of active ingredient (creme nonfibrou powder; NEXT-II) (Ryusendo Co. Ltd.) (Group II) |
| 7 | [60] | Robenacoxib 1 mg/kg/day (Onsior®) (Group I), UC-II® 10 mg/day of active ingredient, Omega 3-fatty acid, Vitamin E (Flexadin® Advanced, Vetoquinol S.r.l.) (Group II) |
| 8 | [61] | Undenatured Type II Collagen 10 mg/day (no specify commercial product). |
| 9 | [62] | UC-II® 10 mg/day of active ingredient (Lonza Consumer Health Inc.) (Group II) |
| 10 | [63] | UC-II® 10 mg/day of active ingredient (Lonza Consumer Health Inc.) (Group II) |
| 11 | [64] | UC-II® 10 mg/day of active ingredient, Omega 3-fatty acid, Vitamin E (Flexadin® Advanced, Vetoquinol S.r.l.) |
| 12 | [65] | Cimicoxib 2 mg/kg/day (Cimalgex®, Vetoquinol) (Group II), UC-II® 10 mg/day of active ingredient, Omega 3-fatty acid, Vitamin E (Flexadin® Advanced, Vetoquinol S.r.l.) (Group III), Cimicoxib 2 mg/kg/day + UC-II® 10 mg/day of active ingredient, Omega 3-fatty acid, Vitamin E (Group IV) |
| 13 | [66] | UC-II® 10 mg/day of active ingredient (Lonza Consumer Health Inc.) (Group II) |
| 14 | [67] | UC-II® 10 mg/day of active ingredient (Lonza Consumer Health Inc.) (Group II) |
| 15 | [34] | *Boswellia serrata* 400 mg/10 kg/day (extract BSB108, BOGAR AG) |
| 16 | [68] | *Boswellia serrata, Harpagophytum procumbens, Ribes nigrum, Salix alba, Tanacetum parthenium,* Omega 3-fatty acid (Group II-A)<br>*Boswellia serrata, Harpagophytum procumbens, Ribes nigrum, Ananas comosus, Curcuma longa,* Omega 3-fatty acid, Glucosamine, Methylsulfonylmethane, Chondroitin, L-glutamate, Hyaluronic acid (Natural Health product formulation) (Group-B) |

| | | |
|---|---|---|
| 17 | [52] | *Boswellia serrata,* Glucosamine, Chondroitin, Chitosamine, Omega- 3/6 fatty acid, green-lipped mussel, *Harpagophytum procumbens* (Sanypet Forza 10, USA Corporation) (Group II) |
| 18 | [26] | *Boswellia serrata,* Glucosamine, Chondroitine, *Ribes nigrum,* Krill flour 1%, *Lentinus edode, Equisetum arvense, Curcuma longa, Harpagophytum procumbens* (Daynamopet srl) (Group II) |
| 19 | [69] | *Boswellia serrata,* Eggshell, Astaxanthin, Hyaluronic acid, Vitamin D (Movoflex, Virbac) (Group II) |
| 21 | [54] | *Boswellia serrata 150 mg/15 kg/day, Cannabis sativa, Cucumis melo L., Cannabidioil* (Candoli Pharma Srl.) |
| 21 | [70] | Glucosamine, Chondroitin, Omega 3-fatty acid, Vitamin C and E, *Saccharomyces Cerevisiae* (Group I)<br>*Boswellia serrata,* Glucosamine, Chondroitin, Omega 3-fatty acid, Vitamin C and E, *Saccharomyces Cerevisiae,* Curcuma (Aurora Biofarma) (Group II) |
| 22 | [71] | *Boswellia serrata,* Canapa oil, *Zingiber officinale,* Vitamin C, Alfa-tocoferol (Evexia Plus, Candoli Pharma Srl.) (Group II) |
| 23 | [72] | *Boswellia serrata Roxb,* Cannabis sativa oil, *Zingiber officinale* extract (Evexia Plus, Candoli Pharma Srl.) (Group II) |
| 24 | [73] | Boswellia gum, Bromelain, Devil's claw tuber, Sarsaparilla root, Dandelion root, Yucca root, Turmeric root extract (Pet Wellbeing Agile Joins) (Group II) |
| 25 | [36] | *Boswellia serrata 31,5 mg,* Undenatured Type II Collagen 4 mg, *Curcuma longa,* Green tea extract, Glucosamine, Chondroitin sulfate, Hyaluronic acid (Confis Ultra, Candioli Pharma Srl) |
| 26 | [37] | *Boswellia serrata*, Undenatured Type II Collagen, Chlorophyll, Green tea extract, Glucosamine, Chondroitin, Hyaluronic acid (Confis Ultra, Candioli Pharma Srl) (Group II) |
